# Integrating Halloysite Nanostraws in Porous Catalyst Supports to Enhance Molecular Transport

**DOI:** 10.1021/acsanm.1c01678

**Published:** 2021-08-06

**Authors:** Oluwole Ajumobi, Yang Su, Azeem Farinmade, Lei Yu, Jibao He, Julia A. Valla, Vijay T. John

**Affiliations:** † Department of Chemical & Biomolecular Engineering, Tulane University, 6823 St. Charles Avenue, New Orleans, Louisiana 70118, United States; ‡ Coordinated Instrumentation Facility, Tulane University, 6823 St. Charles Avenue, New Orleans, Louisiana 70118, United States; § Department of Chemical & Biomolecular Engineering, University of Connecticut, Storrs, Connecticut 06269, United States

**Keywords:** MCM-41, halloysite nanotubes, diffusion, reaction kinetics, mass transfer, effectiveness factor

## Abstract

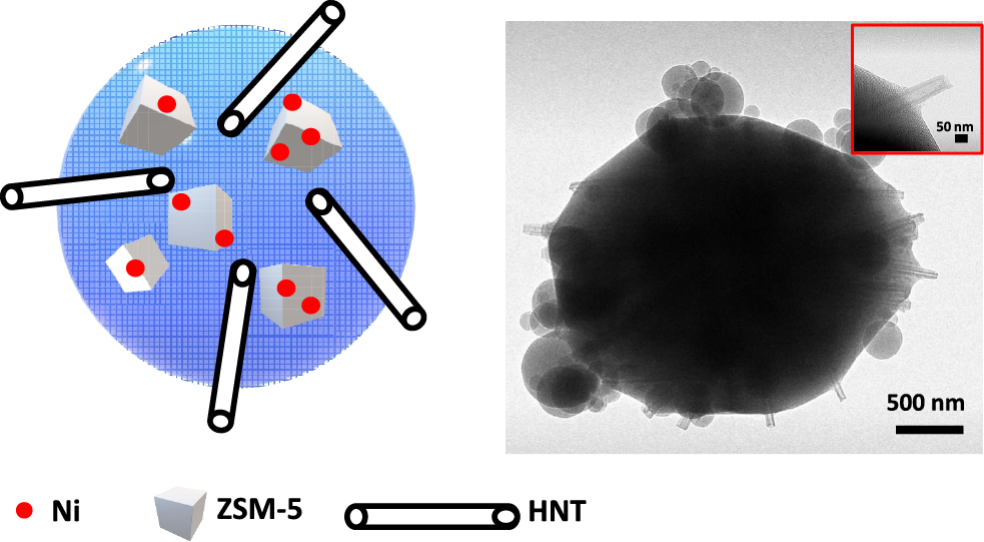

In many porous catalyst supports, the accessibility of interior catalytic sites to reactant species could be restricted due to limitations of reactant transport through pores comparable to reactant dimensions. The interplay between reaction and diffusion in porous catalysts is defined through the Thiele modulus and the effectiveness factor, with diffusional restrictions leading to high Thiele moduli, reduced effectivess factors, and a reduction in the observed reaction rate. We demonstrate a method to integrate ceramic nanostraws into the interior of ordered mesoporous silica MCM-41 to mitigate diffusional restrictions. The nanostraws are the natural aluminosilicate tubular clay minerals known as halloysite. Such halloysite nanotubes (HNTs) have a lumen diameter of 15–30 nm, which is significantly larger than the 2–4 nm pores of MCM-41, thus facilitating entry and egress of larger molecules to the interior of the pellet. The method of integrating HNT nanostraws into MCM-41 is through a ship-in-a-bottle approach of synthesizing MCM-41 in the confined volume of an aerosol droplet that contains HNT nanotubes. The concept is applied to a system in which microcrystallites of Ni@ZSM-5 are incorporated into MCM-41. Using the liquid phase reduction of nitrophenol as a model reaction catalyzed by Ni@ZSM-5, we show that the insertion of HNT nanostraws into this composite leads to a 50% increase in the effectiveness factor. The process of integrating nanostraws into MCM-41 through the aerosol-assisted approach is a one-step facile method that complements traditional catalyst preparation techniques. The facile and scalable synthesis technique toward the mitigation of diffusional restrictions has implications to catalysis and separation technologies.

## 1 Introduction

**Figure 1:**
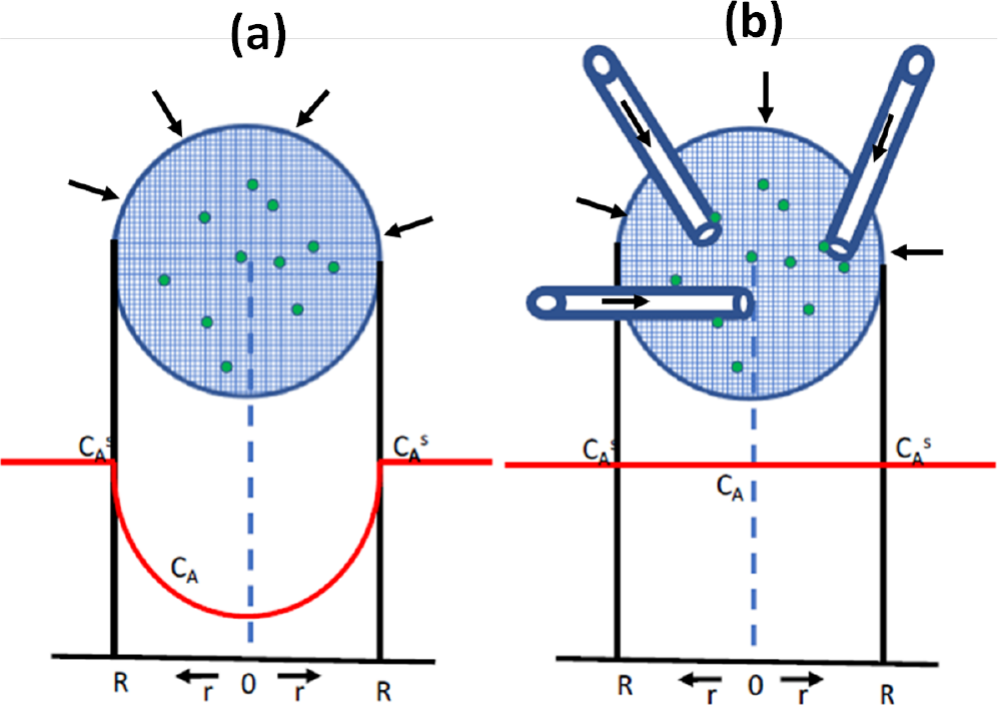
Schematic of the diffusion of reactant species into a spherical pellet (a) with a reduced concentration in the interior and (b) the introduction of straws allows concentrations close to the external surface concentration and enhanced access to interior catalytic sites.

The interplay between reaction and diffusion in porous catalyst particles is a fundamental and foundational concept that impacts the observed reaction rates and selectivities of a host of industrially relevant reactions. In the simplest terms, the role of diffusion for a first-order reaction is described through the Thiele modulus φ = *L*(k/*D*_e_)^1/2^ and the effectiveness factor η = tanh(φ)/φ (slab geometry), where *k* is the intrinsic first-order rate constant, *D*_e_ is the effective diffusivity, and *L* is a characteristic length (half-width of a slab pellet or *R*/3 of a spherical pellet, where *R* is the pellet radius). The concentration of a reactant A in a reaction following first-order kinetics is  (slab geometry) or  (sphere geometry), where  is the concentration of A at the external surface of the particle. Thus, high Thiele moduli values contribute to effectiveness factors significantly below unity and represent catalyst particles where active sites in the interior are exposed to low concentrations of the reactant, thus reducing the observed reaction rate. Figure 1a shows a simple schematic of the concentration profile of a diffusion-limited catalyst particle.

There are many ways to reduce the impact of low catalyst effectiveness factors including modifying catalyst morphologies by placing the active sites near the external surface (the egg-shell model), reducing the particle size, and increasing the pore size of the pellet. These approaches could be cumbersome in practice and could lead to the loss of structural integrity, increased pressure drops in packed beds, etc. We address the problem to find a way to access interior catalytic sites without changing the structural properties of the catalyst particle. We seek to do this through the use of natural clay nanotubes that are inserted into the particle, thus creating the possibility of “nanostraws” to facilitate access to the particle interior. As shown schematically in Figure 1, the insertion of nanotubes into porous particles, where the tube radius is significantly larger than the pore size of the particle, will allow enhanced transport of the reactant into the particle and thus allow enhanced reactant concentrations in the interior, as shown in the idealized schematic of Figure 1, leading to higher effectiveness factors.

The porous particles we focus on are the ordered mesoporous silicas known as MCM-41,^1^ which have significant applications in catalysis^2,3^ and separation technologies.^4,5^ The nanostraws are natural clay nanotubes known as halloysite nanotubes (HNTs). Halloysite is a naturally occurring two-layered aluminosilicate similar to kaolinite, but with a hollow tubular structure caused by the lattice mismatch between the two different layers of clay sheets that leads to the curling of sheets into scrolls. The anionic external surface of a HNT is made up of Si–O–Si tetrahedra, while the internal surface of the lumen consists of Al–OH octahedra with a net cationic charge.^6,7^ The hollow tubular structures of HNTs have a length of 0.5–3 μm, an external diameter of 50–100 nm, lumen diameters varying from 15 to 30 nm, and relatively low surface areas of 22–81 m^2^/g.^8−10^ The silanol external surface of HNTs allows modification with polymers^11,12^ and organosilanes^13^ and can be used as a support for metal nanoparticles.^14,15^ The lumen can be used to load a variety of compounds including drugs and surfactants.^16−18^ Of key importance is the lumen diameter of 15–30 nm, which is significantly higher than the 2–4 nm pore size of MCM-41. Thus, HNTs when inserted into particles of MCM-41 would allow easier access of molecules that approach the size of the pores of MCM-41. To our knowledge, the concept of introducing nanotubes into porous particles to alleviate diffusional restrictions has not been described in the literature, although an important recent paper by Glotov et al.^19^ described the solution phase synthesis of MCM-41 around HNTs to increase the structural rigidity of MCM-41.

**Figure 2:**
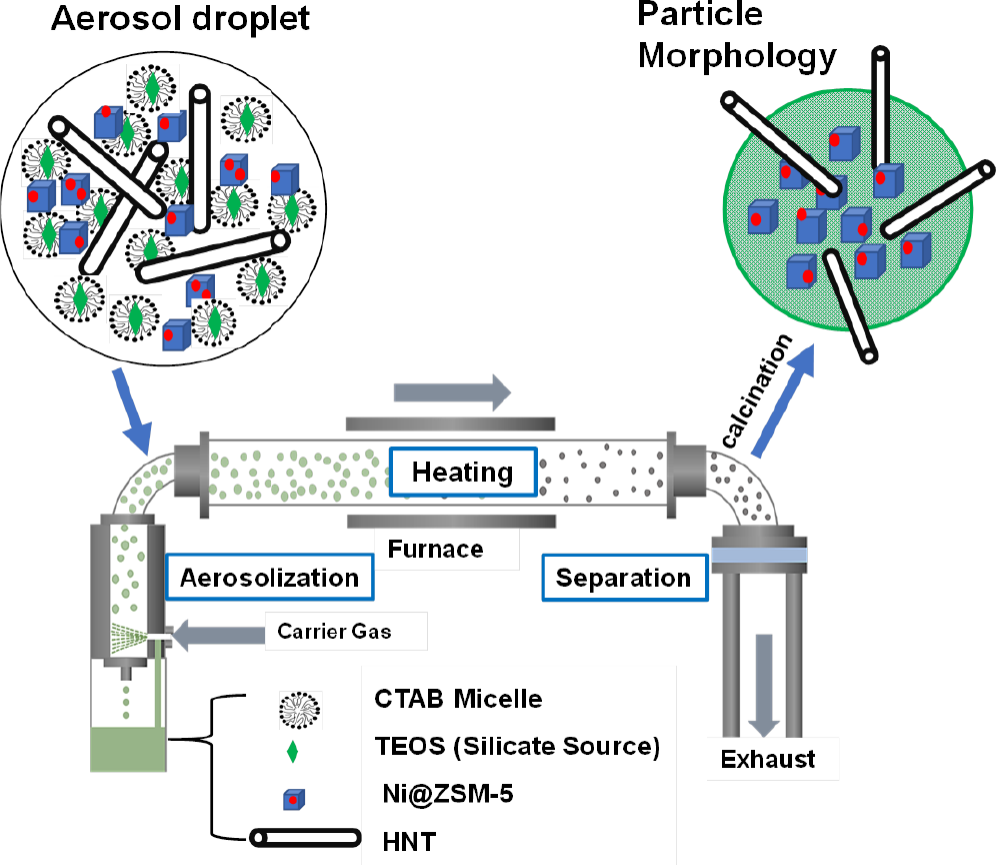
Schematic illustration of the aerosol-assisted synthesis technique. Adapted from Su et al.^21^

The process of inserting such straws into porous particles is a one-step aerosol-assisted process, as shown in Figure 2. The aerosol process for MCM-41 synthesis was first pioneered through the work of Lu et al.^20^ In this process, the precursors including the templating cationic surfactant cetyltrimethylammonium bromide (CTAB) and tetraethoxysilane are dissolved in an ethanol–water solution, which is then passed through an aerosolizer nozzle to produce droplets that are transported through a heated zone. During passage through the heated zone (a tube furnace operated at around 400 °C), silica hydrolysis and condensation occur in each droplet with CTAB templating the formation of ordered mesoporous MCM-41. Each droplet acts as a microreactor where silica synthesis occurs in the confined volume of a droplet with the formation of a single particle from each droplet. Since the external surface of the droplet is in contact with the heated gas, silica synthesis proceeds inward within the droplet as the solvent evaporates.^20−22^ The inherent nature of the aerosol process results in the polydispersity of the particle size, but the fidelity of MCM-41 synthesis is maintained in each particle.^20^

**Scheme 1:**
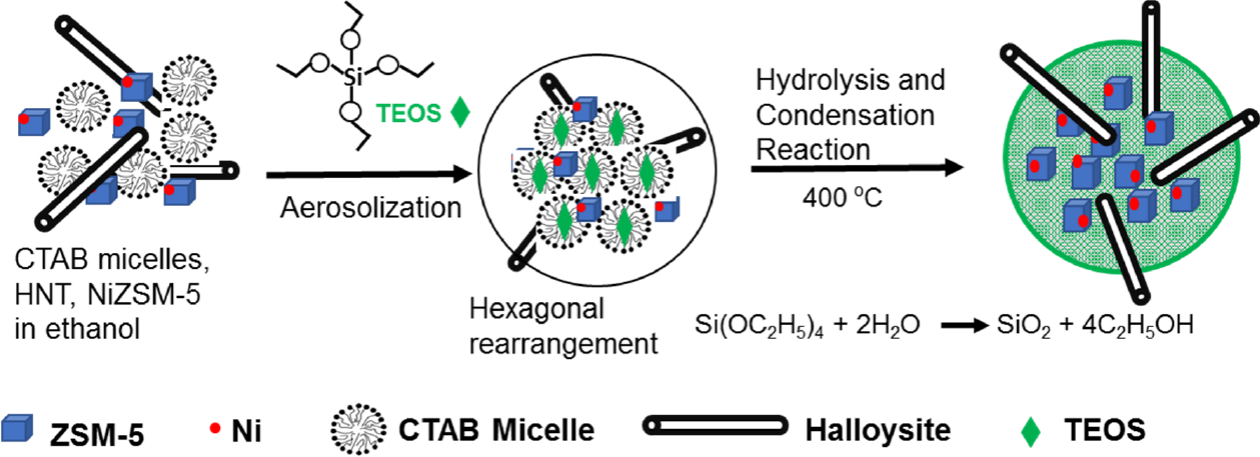
Illustrative Chemistry of MCM-41 Synthesis and the Inclusion of HNTs

Our process is a simple extension of the remarkable finding of Lu et al.^20^ We simply add HNTs to the precursor solution, with the consequence that the HNTs that are much smaller than the 1 mm nozzle size are also captured in the droplets (Scheme 1). As MCM-41 formed, our hypothesis was that the HNTs in the confined volume of the droplet will become entrapped in the solid particles that are formed and if the particles are smaller than the length of the HNTs, it is possible that a portion of the HNTs will extend out of the particle. Thus, this is the concept of the straws to access particle interior.

There is one additional aspect that we have used in our work regarding the actual catalytic sites. Rather than simply including a metal salt into the precursor solution or introducing metal sites after catalyst preparation, we have included microcrystallites of ZSM-5 with Ni clusters on the external surface (Ni@ZSM-5), which are the active sites for the model reaction of the reduction of nitrophenol used in this work.^23^ We do this for the reason that the zeolite microcrystallites are larger than the HNT lumen and therefore have to reside in the matrix of MCM-41. Alternate ways of introducing the metal will result in the metal being deposited in the lumen and will not lead to a direct comparison of the reaction with and without the straws that is necessary for this study, although perhaps inconsequential for application. Our earlier work on integrating zeolites into the matrix of MCM-41^23,24^ indicates that the aerosolization process is entirely feasible for this objective, and the current work expands on this concept to introduce halloysites with the objective of improving access to interior catalytic sites. The following sections of this paper address this focused objective using the liquid phase reduction of nitrophenol as a model reaction.^25^

## 2 Experimental Section

### 2.1 Materials

Tetraethyl orthosilicate (TEOS, 98%), hexadecyltrimethylammonium bromide (CTAB, 95%), nickel(II) nitrate hexahydrate (Ni(NO_3_)_2_·6H_2_O), hydrochloric acid (HCL, 37%), 4-nitrophenol (10 mM), and sodium borohydride (NaBH_4_) were purchased from Sigma-Aldrich and used without any modifications. Nanosized H-ZSM-5 was purchased from ACS Material, LLC (CAS no. 1318-02-1). Camel-lake Australian HNTs (average length of 1 μm and lumen inner diameter of 22 nm) from Australia were received as a gift from John Keeling^26^ (Department of State Development–Geological Survey, South Australia). Ultra Hallopure HNTs were also obtained from I-Minerals, Inc., USA. Deionized (DI) water with a resistance of 18.2 MΩ was remotely obtained from an Elga water purification system (Medica DV25).

### 2.2 Material Synthesis and Encapsulation in MCM-41

The impregnation of 5 wt % Ni on crystallites of ZSM-5 (Ni@ZSM-5) was done via the incipient wetness technique. Typically, 0.5 g of nickel(II) nitrate hexahydrate (Ni(NO_3_)_2_·6H_2_O) salt was dissolved in water and added dropwise to 1.9 g of ZSM-5 powder kept under incipient wetness conditions to obtain the loading of 5 wt % Ni. The resulting solution was dried at 75 °C for 12 h and calcined at 550 °C for 2 h.

The encapsulation of Ni@ZSM-5 in MCM-41 to get a 30 wt % ZSM-5 loading in MCM-41 (M30NZ) was achieved following the procedure in our previously published work.^20^ Briefly, 0.55 g of CTAB was added to 7.5 mL of ethanol and sonicated for 3 min in a bath sonicator (Cole-Parmer 8890). Ni@ZSM-5 (0.12g) was added to the CTAB solution and bath-sonicated for 20 min to allow homogenous dispersion in solution. Under magnetic stirring, 2.25 mL of TEOS was added dropwise followed by the addition of 1 mL of 0.1 M HCl. The resulting precursor solution was immediately transferred into an inexpensive nebulizer (Micro Mist, Teleflex, Inc., 1 mm jet hole diameter) for atomization into aerosol droplets, with the schematic of the aerosol process shown in Figure 2. Using N_2_ as the carrier gas, the droplets were transported into the heating chamber at a flow rate of 2.5 L/min. We note that the carrier gas is bubbled through the precursor solution to form the droplets that are passed through the orifice of the aerosolizer. The heating compartment consists of a quartz tube of 120 cm length with an internal diameter of 5 cm inserted into a furnace of 76 cm length that is operated at 400 °C. A short residence time of approximately 36 s was achieved based on the dimensions of the furnace and the flow rate of the carrier gas. Dried and powdered particles from the heating zone were collected on a cellulose filter paper (Merck Millipore Ltd., pore size = 0.22 μm) maintained at 80 °C by heating tapes to prevent moisture condensation. The collected particles were calcined in air at 550 °C (heating rate of 5 °C/min) to completely remove the surfactant template.

The integration of HNTs into MCM-41 containing Ni@ZSM-5 (M30NZ/30HNT) was accomplished by adding HNTs into the precursor solution of TEOS and zeolite microcrystals. The amounts of precursor species are modified to achieve 30 wt % each of Ni@ZSM-5 and HNTs in the composite. Thus, 0.425 g of Ni@ZSM-5 (30 wt %) was added to the CTAB solution (1.1 g of CTAB in 15 mL of ethanol) and bath-sonicated for 20 min to achieve even dispersion in the solution. HNTs (0.425 g, 30 wt % based on silicon composition in TEOS) were added to the obtained suspension of Ni@ZSM-5 in CTAB solution, and the solution was vigorously stirred for 1 h to evenly disperse HNTs. Afterward, 4.5 mL of TEOS was added dropwise followed by the addition of 2 mL of 0.1M HCl after 3 min. The resulting mixture was aerosolized following the same procedure described above with the slight variation of increasing the flow rate to 4 L/min (22 s residence time) simply to enhance the bubbling rate through the precursor solution to keep the HNTs continually suspended.

### 2.3 Material Characterization

The composite particles were characterized through X-ray diffraction (XRD) and BET surface area analysis, and their morphologies were examined using electron microscopy. Structural properties of the samples were obtained via XRD (Rigaku Miniflex II with a Cu Kα radiation at 1.54 Å) at a scanning range of 2θ = 1.5–7.0° for small-angle scanning and up to 80° for wide-angle scanning at room temperature. Porosity and surface area were evaluated via the nitrogen gas sorption technique (Micromeritics, ASAP 2010) using the Brunauer–Emmett–Teller (BET) isotherm for the evaluation of surface area and the Barrett–Joyner–Halenda (BJH) analysis for the estimation of pore volume. Morphological characterizations were done through imaging using scanning (SEM, Hitachi SEM-4800 field emission operated at 3 kV) and transmission (TEM, FEI Tecnai G2 F30 twin transmission operated at 300 kV) electron microscopy. Cut-section TEM samples were prepared by embedding the particles within epoxy resin followed by making thin sections (100 nm) using a diamond knife. Preparation of samples for cut-section SEM followed the same procedure as that of fixing the sample in epoxy resin, but only one cut was made to generate a thick section that could be easily mounted on an SEM stub. For SEM, the samples were carbon-coated prior to imaging.

### 2.4 Catalytic Application of Composite Particles

To confirm the accessibility of reactants to encapsulated Ni@ZSM-5 through pores of MCM-41 and the effect of HNTs acting as straws for improved diffusion, we tested the catalytic performance of the composite samples for the reduction of 4-nitrophenol (4-NP) to 4-aminophenol (4-AP) as a model reaction.^25,27^ Typically, 15 mL of 0.1M NaBH_4_ was added to 5 mL of 0.1mM 4-nitrophenol in a 40 mL glass vial and stirred. A weighed amount of catalyst Ni@ZSM-5 or composite samples (M30NZ and M30NZ/30HNT) was added to the mixture, and the reaction was initiated. To monitor the reaction, 1.7 mL of the reactant mixture was sampled at various time intervals and analyzed using UV–vis spectroscopy (Shimadzu UV-1700 PharmaSpec). The sampled reactants were removed from the UV–vis spectrophotometer and transferred into the vial after analysis to preserve the catalysts. The addition of excess NaBH_4_ to 4-nitrophenol as a reducing agent leads to the formation of 4-nitrophenolate, which is a reaction intermediate, and the presence of active metal sites allows the reduction of activation energy, which promotes the formation of 4-aminophenol.^28^ Gradual reduction of the absorbance peak of 4-nitrophenolate at 400 nm and concurrent appearance of the increasing 4-aminophenol peak at 300 nm are monitored. The complete reaction is achieved when the 4-nitrophenolate peak entirely disappears.

## 3 Results and Discussion

### 3.1 Synthesis and Characterization of MCM-41 Containing HNTs and Zeolite Microcrystals

**Figure 3:**
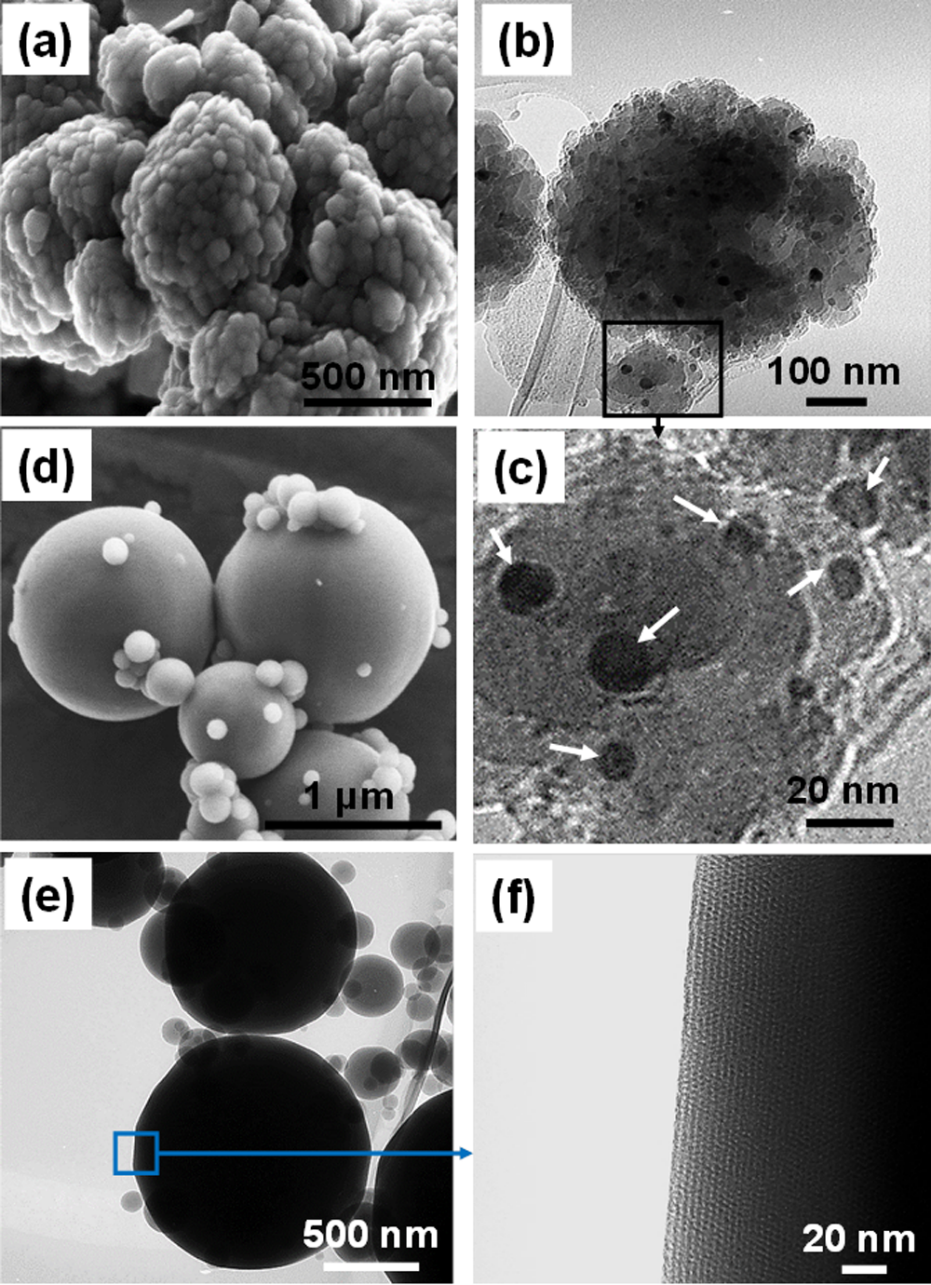
(a) SEM images of Ni@ZSM-5 showing ZSM-5 particles (primary size: 50–100 nm). (b) TEM image of Ni@ZSM-5. (c) High-resolution TEM image showing the dispersion of Ni nanoparticles on the surface of ZSM-5 as dark spots. (d) SEM image showing the spherical morphology of M30NZ with polydispered particle size. (e) TEM image of M30NZ. (f) High-resolution image of M30NZ showing the ordered array of uniform pores.

The step-by-step approach to the implementation of the concept starts with the synthesis of MCM-41 containing 30 wt % of Ni@ZSM-5 (M30NZ) using the aerosol-assisted synthesis technique.^23^Figure 3a shows the SEM image of Ni-impregnated ZSM-5 with primary particles of sizes of 50–100 nm that are aggregated together.^22,29^ The TEM image of Ni@ZSM-5 in Figure 3b shows 5–10 nm clusters of the metal atom on the surface of ZSM-5 with better visualization in the high-resolution TEM shown in Figure 3c. The obtained Ni@ZSM-5 was added to the MCM-41 precursor solution containing TEOS and CTAB and allowed to stir vigorously to maintain suspension stability during aerosolization. Aerosolization results in the formation of Ni@ZSM-5 encapsulated in the mesoporous matrix of MCM-41. The terminology M30NZ is used to denote the MCM-41 matrix containing 30 wt % Ni@ZSM-5.

The SEM and TEM images of composite particles of M30NZ are shown in Figure 3d–f. The images reveal the spherical morphologies similar to those of pure MCM-41 formed through the aerosol process (SEM and TEM, section S1). On examining Figure 3d, we observe that there is a wide distribution of particle size. The large particles that are of the order of 1 μm are those that contain ZSM-5 and emanate from droplets that contain zeolite microcrystallites. The small particles in the range of 100 nm are those that appear to be formed from the droplets that do not contain zeolite and are therefore purely MCM-41. Figure 3e shows the corresponding TEM of a distribution of particles, and the high-resolution TEM of Figure 3f illustrates the ordered morphology of pores of MCM-41 that can be observed through direct TEM imaging. The fact that no separate crystallites of ZSM-5 are observed on the SEMs or the TEMs implies the complete encapsulation of ZSM-5 in the large particles of MCM-41. Our earlier papers have shown cut-section TEMs of these particles to provide evidence of zeolite encapsulation,^23,24^ and we do not elaborate here since the incorporation of HNTs is the main objective of this work.

**Figure 4:**
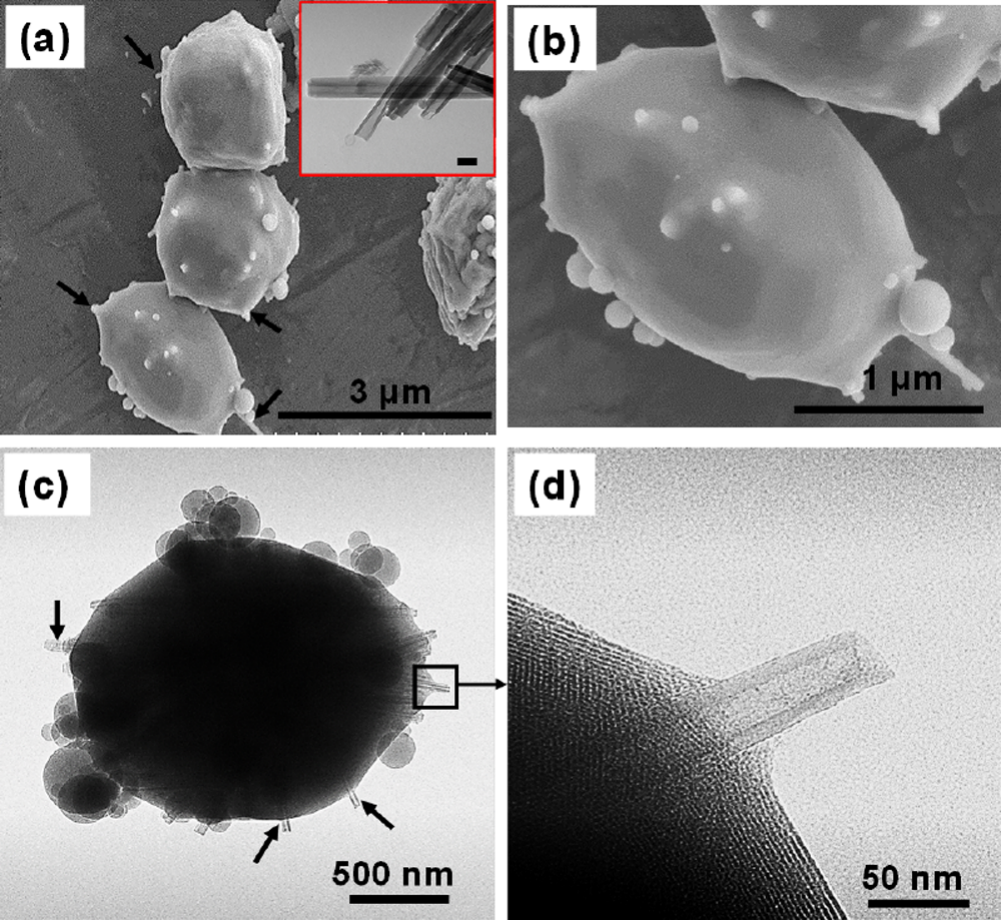
SEM images of (a) M30NZ/30HNT showing HNTs (black arrows) protruding as straws. The inset is a TEM image of pristine HNTs; scale bar = 50 nm. (b) The higher-resolution image showing additional details of large particles with satellite small spherical particles. (c) TEM image of M30NZ/30HNT showing HNT protrusions from a catalyst particle. (d) High-resolution TEM image showing a clear lumen of the protruding HNT.

The key aspect of our work is the incorporation of HNTs into these particles. This is simply done by adding HNTs into the precursor system together with the microcrystallites of ZSM-5 and carrying out the aerosolization, as shown in Figure 2. The concept is very simple that hydrolysis and condensation of MCM-41 in the confined volume of the droplet will result in both encapsulation of the 200 nm Ni@ZSM-5 particles and integration of the HNT in the MCM-41 matrix. The results are shown in Figure 4, and the terminology M30NZ/30HNT represents the encapsulation of 30 wt % Ni@ZSM-5 in MCM-41 coupled with the incorporation of 30 wt % HNTs. The presence of CTAB leads to the formation of an ordered mesoporous matrix of MCM-41, which entraps Ni@ZSM-5. Since HNTs are present in the aerosol droplet, they become integrated into the silica matrix and part of their length protrudes from the mesoporous silica spheres. As schematically illustrated in Figure 2, calcination of the collected dry product consequently leads to a final structure of Ni@ZSM-5 microcrystallites encapsulated within the mesoporous matrix of MCM-41 and HNTs protruding as straws.

Figure 4a,b shows the SEM images of composite particles of M30NZ/30HNT. We observe spherical elongated micron-sized particles together with spherical satellite particles of the order of 100–200 nm that are simply MCM-41. No zeolite microcrystallites are observed in the SEMs again, indicating the full encapsulation of the zeolite, while the protuberances are the HNTs. We also note that there is no evidence of HNTs present outside the particles, indicating that all HNTs have been integrated into the MCM-41 matrix, many with their ends protruding from the particles. We attribute the nonspherical morphology of the composite to the presence of HNTs. The adsorption of CTAB on the anionic exterior of HNTs serves to nucleate the formation of MCM-41 as hydrolysis of TEOS leads to anionic intermediates that are templated by the cationic surfactant. As the evaporating front recedes inward, a film of MCM-41 is formed first on the adsorbed CTAB, followed by rapid propagation of MCM-41 to the bulk of the fluid in the droplet. The SEM and TEM images of Figure 4 indicate this effect, as shown by the arrows of meniscus-shaped mesoporous silica around the protruding section of the HNTs in Figure 4c,d. The protrusions of multiple HNTs from a single particle can be seen in Figure 4c. Figure 4d is a high-resolution micrograph of one of the HNTs, and it is immediately seen that the 20 nm lumen appears clear in the protruding region. Indeed, all of the protruding nanotubes in Figure 4c appear to have clear lumens in the region that is visible through direct TEM imaging. This is an important observation as it relates to the transport of species through these straws to the interior. The lumen thus serves as a larger channel for the transport of reactant species toward the active core as well as the transport of product molecules out of the particle. Our reason for the empty lumen is simply based on the fact that the interior of the lumen is cationic with a zeta potential value of +24 mV for the lumen,^30−32^ thus inhibiting the adsorption of CTAB. Capillary effects rapidly pull micelles of CTAB containing TEOS to the internal end of the tube where they then form MCM-41. We cannot discount the possibility that MCM-41 will form at the internal ends of the HNTs but posit that this will still reduce the diffusional restrictions and will allow access to internal catalytic sites.

**Figure 5:**
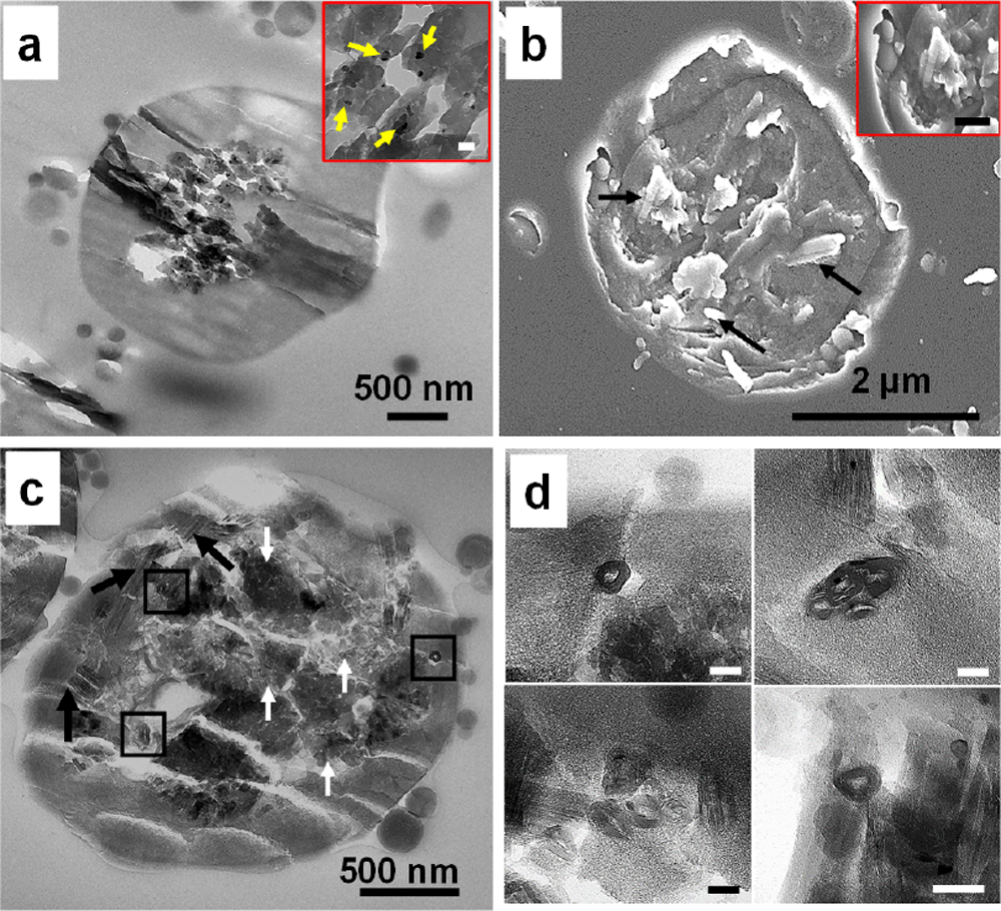
(a) Cut-section TEM of M30NZ showing the presence of incorporated Ni@ZSM-5 within the mesoporous matrix. The inset is a high-resolution image showing Ni nanoparticles (yellow arrows) as black dots on ZSM-5; scale bar = 50 nm. (b) Cut-section SEM image of M30NZ/30HNT showing the presence of HNTs inside the composite (black arrows). (c) M30NZ/30HNT showing the encapsulated Ni@ZSM-5 (white arrows) and different orientations of HNTs lying parallel to the cut section (black arrow) and orthogonal to the cut section (black boxes). (d) Collage of high-resolution images showing HNTs with evidence of a clear lumen; each scale bar = 50 nm.

Cut-section SEM and TEM images of M30NZ and M30NZ/30HNT in Figure 5 provide evidence of zeolite encapsulation within the MCM-41 matrix and the simultaneous incorporation of HNTs as tubular straws. The cut-section TEM image in Figure 5a shows the presence of zeolite microcrystals with dark spots of the electron-dense Ni indicating encapsulation of Ni@ZSM-5. We note that our earlier work^23^ has expanded on this process with the EDS results on the cut section to quantify the elemental composition. Figure 5b shows a cut-section SEM of M30NZ/30HNT, which was simply done by cutting the sample fixed in epoxy just once prior to mounting on the SEM holder (in contrast, for TEM imaging, the epoxy-fixed sample is cut twice to generate the thin 100 nm section). The cut-section SEM image in Figure 5b shows the presence of trapped HNTs within the composite. This is supported by the TEM cut-section image of M30NZ/30HNT fixed in epoxy shown in Figure 5c. There is clear evidence of both Ni@ZSM-5 (white arrows) and HNTs (black boxes) in the cut sections. We note that the random cut section shows HNTs in different orientations and the black arrows point to HNTs lying somewhat parallel to the cut. However, very interestingly, we are able to capture a few HNTs that may have been cut orthogonal to their orientation showing the lumen as indicated by the black boxes. Figure 5d is a collage of these black boxes at a higher resolution obtained from multiple cut sections. The ability to visualize a lumen fairly clearly is perhaps further evidence of the fact that the lumens are not filled with MCM-41, although they could contain epoxy that may have infiltrated during the cut-section procedure.

**Figure 6:**
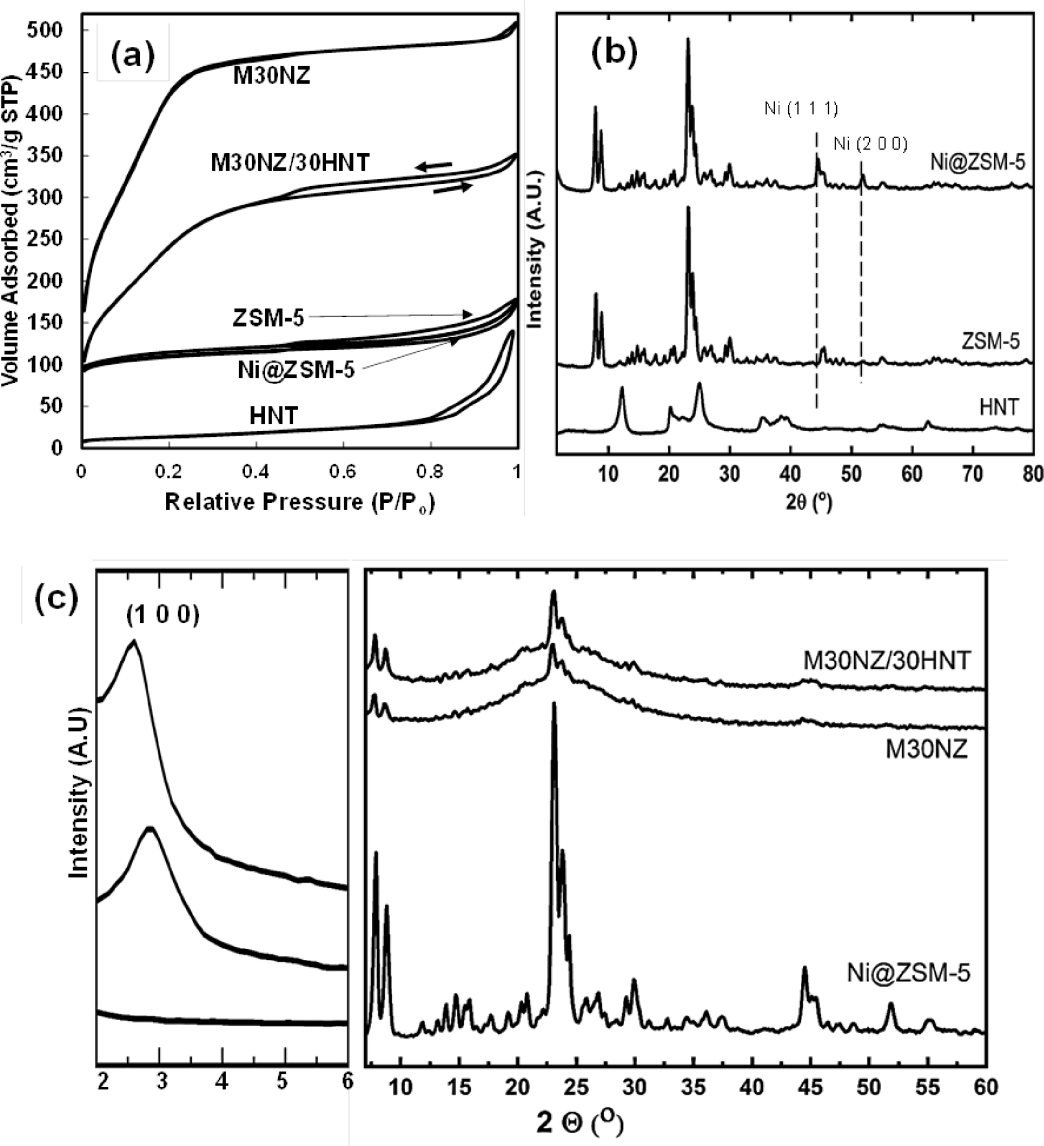
(a) BET isotherm plot for ZSM-5, HNTs, Ni@ZSM-5, M30NZ, and M30NZ/30HNT. (b) XRD of HNTs, ZSM-5, and Ni@ZSM-5 with visible Ni peaks at the (111), (200), and (220) crystal planes. (c) Small- and wide-angle XRD scans of Ni@ZSM-5, M30NZ, and M30NZ/30HNT.

Nitrogen sorption analysis and XRD patterns of zeolite samples and composite particles are shown in Figure 6 and summarized in Table 1. ZSM-5 and Ni@ZSM-5 exhibit BET surface areas of 354 and 340 m^2^/g, respectively, in agreement with the results of Pan et al.^22^ and Lai et al.^33^ with the addition of Ni having a negligible effect on the surface areas of ZSM-5. M30NZ exhibits a surface area of 1391 m^2^/g and a higher pore volume of 0.22 cm^3^/g due to the mesoporous matrix of MCM-41. The high surface area indicates that the encapsulated zeolite crystals within the MCM-41 matrix do not affect the mesopore structure of the MCM-41 matrix. M30NZ/30HNT has a lower surface area of 889 m^2^/g but a higher pore volume (0.35 cm^3^/g) compared to M30NZ. The lower surface area observed is simply based on the normalization of the surface area per gram of the composite combining the high surface areas of MCM-41 with the low surface area of the tubular HNT (50 m^2^/g.^8,10,19^ M30NZ/30HNT also shows evidence of a hysteresis loop in the BET isotherm symptomatic of a type IV isotherm.^34,35^ The hysteresis loops in Figure 6a are attributed to the integration of tubular HNT straws of inner diameter 20–30 nm, leading to multilayer adsorption followed by desorption from a meniscus of nitrogen due to capillary condensation. Repeated synthesis with systems containing various loading levels of HNTs (data not shown) all shows evidence of such type IV isotherms. The type IV isotherm obtained by the filling of HNTs by N_2_ is another possible evidence of the tubes being essentially empty, thus allowing capillary condensation. Section S2 provides additional characterization results on integrating other sources of HNTs (i-Minerals) into MCM-41, indicating the generation of type IV isotherms.

**Table 1:** Nitrogen Sorption Analysis

sample	BET surface area (m^2^/g)	pore volume (cm^3^/g)
ZSM-5	354	0.15
Ni@ZSM-5	340	0.14
MCM-41	1429	0.19
M30NZ	1391	0.22
HNT	49	0.21
M30NZ/30HNT	889	0.35

Structural analyses of the zeolite and composites samples through XRD are shown in Figure 6b,c. As shown in Figure 6b, the Ni@ZSM-5 sample exhibits characteristic (111), (200), and (220) diffraction peaks of Ni in addition to the diffraction pattern of crystalline ZSM-5.^23,36^ Both the composite samples of M30NZ and M30NZ/30HNT (Figure 6c) exhibit the characteristic (100) diffraction peak of MCM-41 at 2θ = 2.8° and 2.6°, respectively. These peak positions correspond to the MCM-41 *d*-spacings of 3.2 and 3.4 nm, respectively.^37,38^ We note the increased *d*-spacing in the M30NZ/30HNT system and attribute this to the fact that there are two populations of MCM-41: one that grows directly through CTAB micelle templating and the other that is based on MCM-41 growing on the external surface of HNTs where CTAB adsorbs. Since the CTAB micelle templating is present in both systems of M30NZ and M30NZ/30HNT, the growth of MCM-41 from adsorbed CTAB on the surface of HNT may be the cause of the increased *d*-spacing. The BET surface area, XRD analysis, and imaging data are further evidence of successful encapsulation of Ni@ZSM-5 and HNT into the MCM-41 matrix while preserving the morphology of MCM-41.

### 3.2 Evaluation of Catalytic Activity

**Scheme 2:**
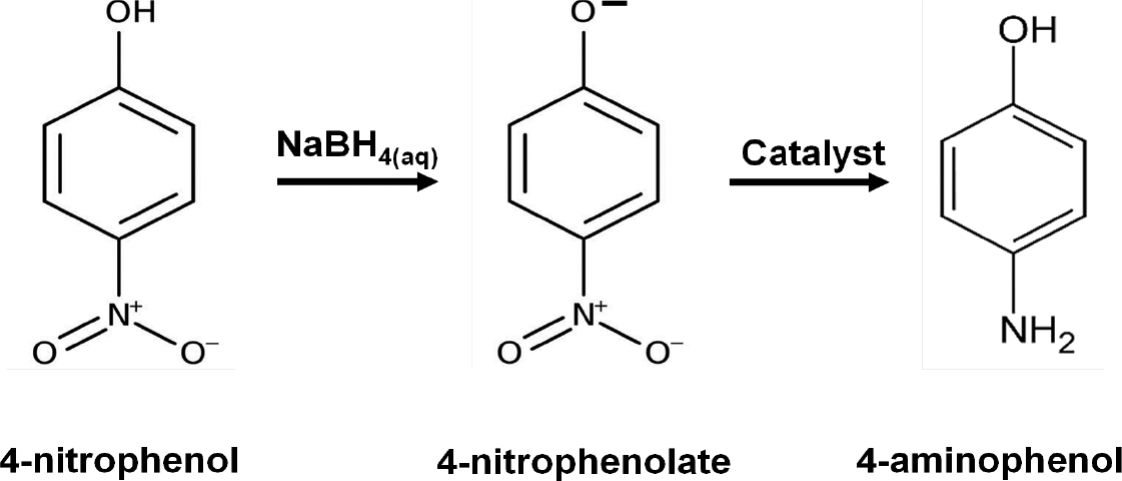
Reaction Mechanism for Catalytic Reduction of 4-Nitropehnol to 4-Aminophenol over Composite Particles

In our recent work, we have shown that zeolite microcrystals embedded within MCM-41 are accessible to reactants^23,24^ although there are diffusional restrictions to transport. The concept of incorporating HNTs as straws to mitigate such diffusional restrictions and enhance the observed reaction rate is the new aspect of the current paper. We therefore used a model reaction, the aqueous phase catalytic reduction of 4-nitrophenol (4-NP) to 4-aminophenol (4-AP) in the presence of excess NaBH_4_ (sodium borohydride), to compare the catalytic activity of M30NZ and M30NZ/30HNT and understand the role of HNT inclusion in the composite (Scheme 2). The kinetic diameter of 4-NP is 0.7 nm, which is not insignificant in comparison to the 2–3 nm pore dimensions of MCM-41 and is larger than the 0.5 nm pore dimensions of ZSM-5. We expect Knudsen type diffusivities in MCM-41, and adsorbed 4-NP and adsorbed water may pose further diffusional restrictions.

**Figure 7:**
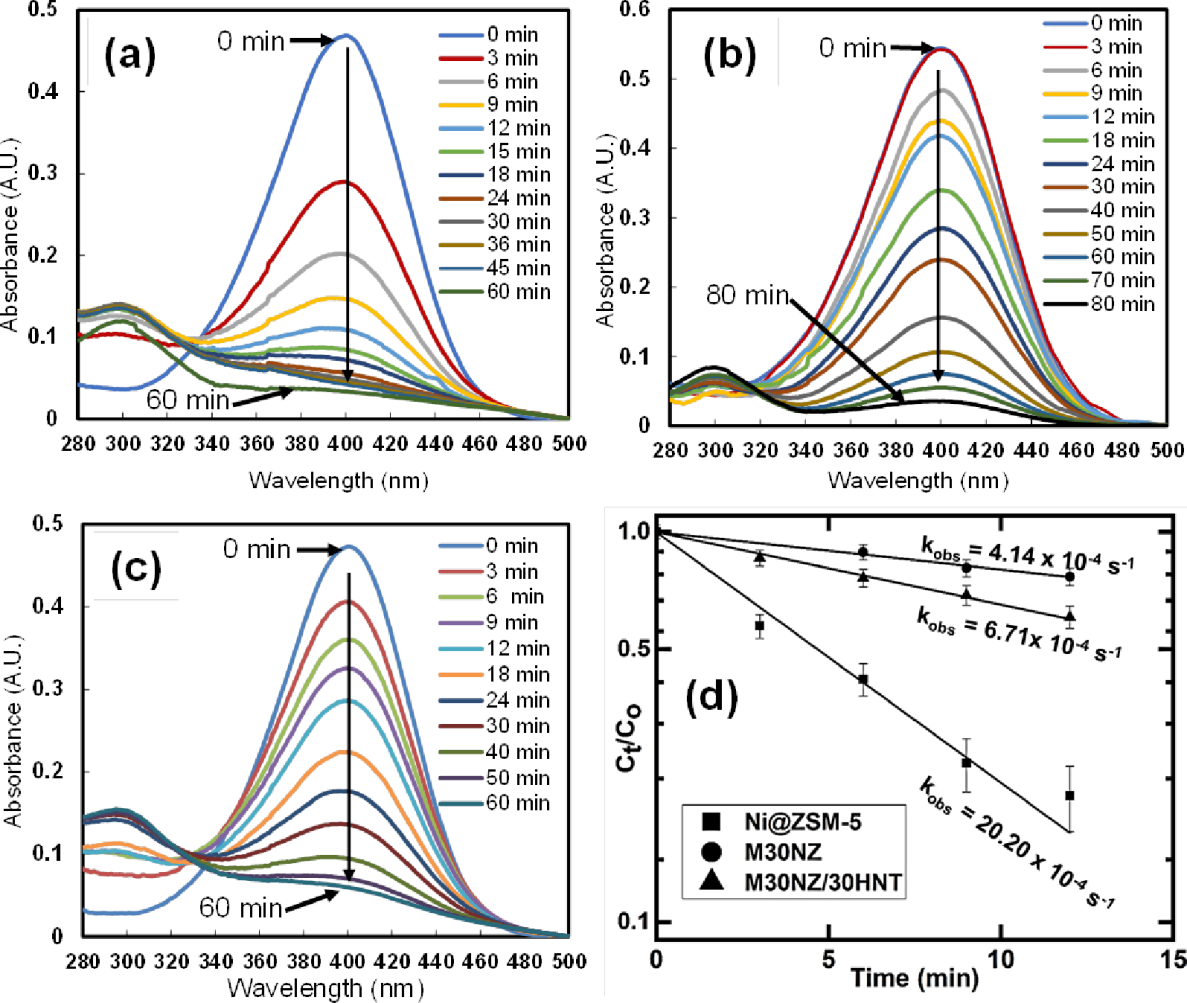
UV–vis spectra showing the reduction of 4-nitrophenol to 4-aminophenol over (a) 2.5 mg of Ni@ZSM-5, (b) 3 mg of M30NZ, and (c) 3 mg of M30NZ/30AHNT. (d) Kinetics of 4-nitrophenol reduction fitted to pseudo-first-order rate constants.

The absorption peak of 4-NP is observed at 320 nm, and the addition of NaBH_4_ leads to the formation of 4-nitrophenolate anions with an absorption peak 400 nm.^39^Figure 7 illustrates the kinetics of 4-nitrophenol reduction in various systems using the Ni@ZSM-5 data as the reference. The kinetics of the reaction are shown in Figure 7d, where the data is fit to a pseudo-first-order reaction, and the observed first-order rate constants are also reported in Figure 7d. When normalized per mass of the catalyst, the rate constants are converted to *k*_obs_ of 0.81 s^–1^/g cat for Ni@ZSM-5 (2.5 mg of catalyst added) and 0.14 s^–1^/g cat for M30NZ (3 mg of catalyst added). The integration of HNTs into the system (M30NZ/30HNT) leads to an improvement in the observed rate constant to 0.23 s^–1^/g cat (3 mg of catalyst added), which is 62% higher than that of M30NZ. The improvement is even more marked when the rates are normalized in terms of the Ni@ZSM-5 component, which is the active component in the composite. Thus, the observed reaction rate constants are 0.46 s^–1^/g for Ni@ZSM-5 and 0.75 s^–1^/g for Ni@ZSM-5 since Ni@ZSM-5 is 30 wt % of the total mass in each of the composites. If we use the pristine Ni@ZSM-5 as the reference system with an effectiveness factor of unity, the relative effectiveness factors reflecting the ratio of the observed rate constant in the composite to the observed rate constant in Ni@ZSM-5 are 0.6 in the M30NZ system and 0.9 in the M30NZ/30HNT system.

With the observed rates of reaction, the concepts of external mass transfer are implicit. We note two aspects, however. First, all systems do incorporate external mass transfer, but the fact that the comparisons are made between different materials implies that the background of external mass transfer is essentially subtracted out in the interpretations and comparisons of the effectiveness factor. Second, the solutions are stirred, which reduces the effects of external mass transfer. Thus, the reduction of 4-nitrophenol to 4-aminophenol over the three catalysts was done under the same experimental conditions, indicating that the key differences are due to the internal transport and reaction.

There are some distinctions in the M30NZ and M30NZ/30HNT catalyst particles. First, they are different catalytic materials as M30NZ contains 30 wt % Ni@ZSM-5 and 70 wt % MCM-41, while the M30NZ/30HNT contains 30 wt % Ni@ZSM-5, 30 wt % HNT, and 40 wt % MCM-41. The addition of HNTs increases the particle size through the inclusion of HNTs, and M30NZ/30HNT has a particle size of 3 ± 0.8 μm, while M30NZ has a particle size of 1.5 ± 0.4 μm. Intrinsically, this implies a larger Thiele modulus for the M30NZ/30HNT system and should lead to a reduced observed reaction rate. The fact that there is still an enhancement in observed reaction rates for the M30NZ/30HNT system with larger particles points to a strong possibility that the system may be one where diffusional restrictions are reduced through the introduction of the HNTs. Indeed, particles without HNTs and composed entirely of MCM-41 are significantly smaller than the particles with HNTs. Conceptually, this simply validates the point that we can generate large particles with larger pores that allow entry and access to the particle interior. The MCM-41 matrix in the large particles stays the same in terms of pore size, so diffusional effects are not expected to be different in MCM-41 in any of the systems. Thus, the enhanced reactivity in the large particles containing HNTs can only be explained by diffusion through the lumen. One can simply think of the system as etching out larger cylindrical pores in large particles of MCM-41 without impacting the structural stability of MCM-41. The relative effectiveness factor of 0.9 in the M30NZ/30HNT system indicates that the composite material is able to function almost as effectively as powdered Ni@ZSM-5 microcrystallites without the attendant difficulties of increased pressure drops, etc., when packed into tubular reactors.

## 4 Conclusions

We have developed a one-step aerosol-assisted method of designing composite catalytic materials with the specific objective of mitigating diffusional limitations in porous catalyst particles. Thus, the integration of HNTs into the porous matrix of MCM-41 facilitates the entry of reactants to the particle interior and access to interior catalytic sites. With the model reaction of the reductive amination of p-nitrophenol, the observed pseudo-first-order rate constant increases from 0.46 s^–1^/g of the active material in the HNT-free system to 0.75 s^–1^/g in the system containing the HNT nanostraws, an increase of 63%. The relative effectiveness factor based on the reference of powdered active material (Ni/ZSM-5) increases from 0.6 to 0.9. A particularly appealing aspect of the material synthesis is that it is essentially a one-step process occurring in the confined environment of an aerosol droplet. The semi-continuous nature of the aerosol process indicates scalability through multiple units operating in parallel. In addition to the incorporation of HNTs, the technique can be adapted to the coencapsulation of zeolite microcrystallites, thus leading to novel composite structures. These observations indicate the viability of introducing HNTs as nanostraws to enhance access to the interior of mesoporous materials synthesized through a “ship-in-a-bottle” approach.

There are several potential implications to the generation of such particle morphologies, and we touch on areas of continuing research. From a catalytic perspective, such materials may allow more rapid access of larger molecules to interior catalytic sites, thus leading to enhanced reaction rates. The concept of integrating zeolites into these high–surface-area mesoporous matrices indicates opportunities for process intensification where catalytic sites on the mesoporous matrix and the zeolite catalytic sites can be coupled as in the bifunctional aspect of catalytic reforming. The high-surface-area matrix can also be used as a sink for catalyst poisons or coke formation in such composites. The protrusions of HNTs imply opportunities to pack reactors with increased spacings between particles, allowing reduced pressure drops even if small particles are used.

## Supporting Information Available

SEM and TEM data for pristine HNTs and bare polydispersed MCM-41 particles (section S1) and morphological characteristics of substitution of camel-lake HNTs with longer I-mineral HNTs in MCM-4 (section S2) (PDF)
