# Supplementary material for: Integrating Halloysite Nanostraws in Porous Catalyst Supports to Enhance Molecular Transport
Source: ACS Appl Nano Mater. 2021 Aug 6;4(8):8455–64. doi: 10.1021/acsanm.1c01678 (PMC8406414; doi:10.1021/acsanm.1c01678)
Supplement: Supplementary file 1 — an1c01678_si_001.pdf [file an1c01678_si_001.pdf]

SUPPORTING INFORMATION

Integrating Halloysite Nanostraws in Porous Catalyst Supports to enhance Molecular Transport

Oluwole Ajumobi[1], *Yang Su*[1], *Azeem Farinmade*[1], *Lei Yu*[3], *Jibao He*[2], *Julia A. Valla*[3], *Vijay T. John*[1*]

1. Department of Chemical & Biomolecular Engineering, Tulane University, 6823 St. Charles Avenue, New Orleans, Louisiana 70118, United States.
2. Coordinated Instrumentation Facility, Tulane University, 6823 St. Charles Avenue, New Orleans, Louisiana 70118, United States.
3. Department of Chemical & Biomolecular Engineering, University of Connecticut, Storrs, Connecticut 06269, United States.

Submitted to *ACS Applied Nano Materials*, July 2021.

*Corresponding authors

Vijay T. John – vj@tulane.edu

Julia A. Valla – Ioulia.valla@uconn.edu

S-1: Morphological Characterization of Pristine Halloysite Nanotubes and Bare MCM-41

The SEM image of pristine HNT in Figure S-1a shows its tubular orientation. From the TEM image in Figure S-1b, the tubular structure of HNT with an average length of 1.0 – 1.5 μm and visible lumen of (20-30 nm) are observed. The SEM images in Figure S-1c reveals the spherical morphology of MCM-41 particles while the TEM (Figure S-1d) shows the ordered mesopore structure.

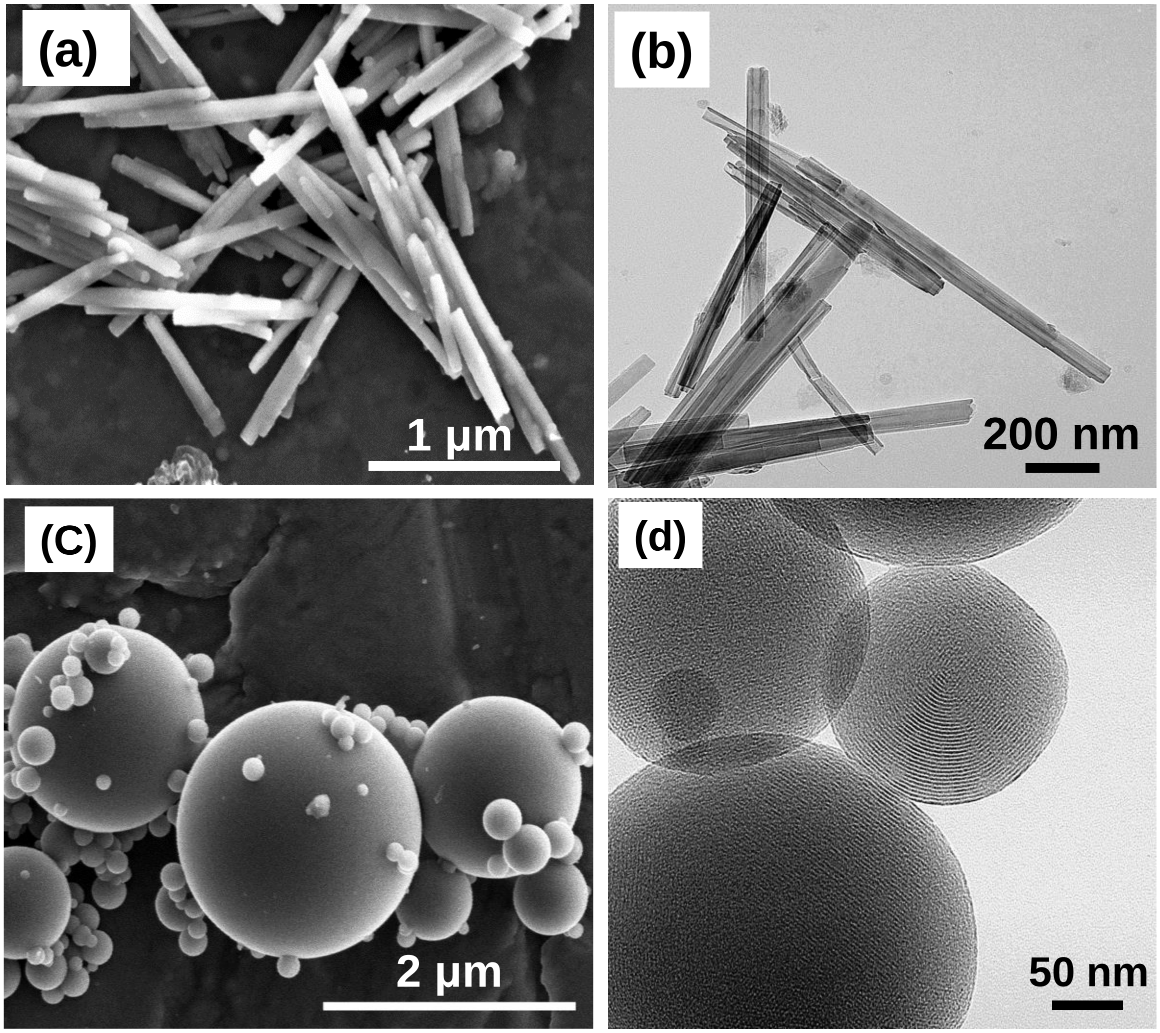

Figure S-1. Electron micrograph showing the morphology of pristine HNT and bare MCM-41 (a) SEM images of camel-lake HNT (b) TEM images of camel-lake HNT (c) SEM image of bare MCM-41 (d) TEM image of bare MCM-41.

S-2: MCM-41 containing longer I-Mineral Halloysite Nanotubes

Figure S-2 shows data on the integration of long HNTs (I-minerals, HNT length 1.5-2 μm) into MCM-41. Figure S-2a shows the characteristic Type IV isotherm, while Figure S-2b and S-2c show morphological deviations from sphericity when the HNT loading is increased from 50 wt% to 75 wt%.

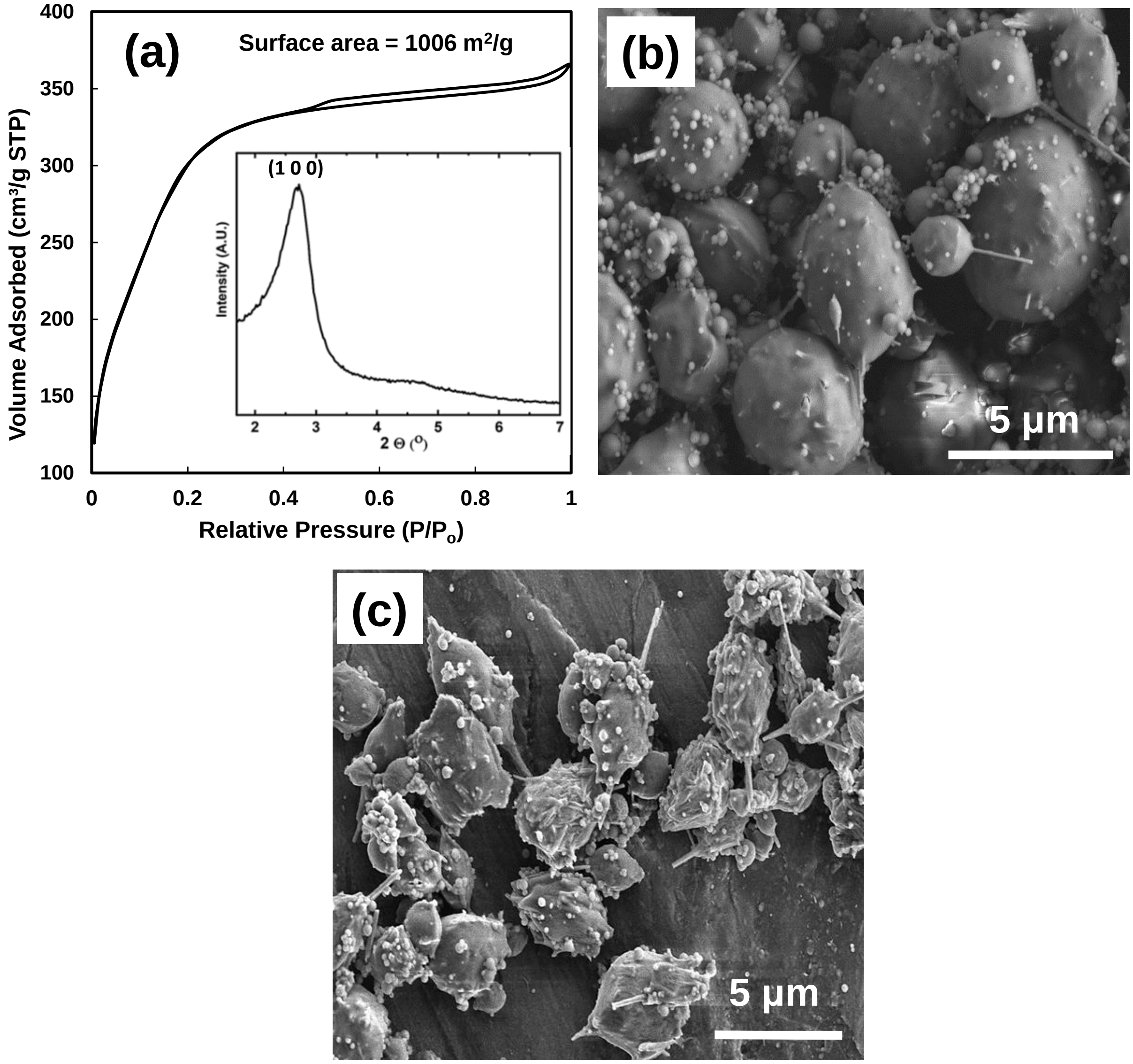

Figure S-2. (a) BET isotherm showing a type IV hysteresis loop. Inset is the XRD profile showing characteristic peaks of MCM-41 in composite sample of 50 wt% I-mineral HNT integrated with MCM-41. (b) SEM image showing the longer length of I-mineral HNT protruding out as straws (c) SEM image of 75 wt% I-mineral HNT in MCM-41.
